# What's being said: exploring parental comments during a family meal session in family based treatment for avoidant/restrictive food intake disorder

**DOI:** 10.3389/frcha.2026.1858934

**Published:** 2026-06-22

**Authors:** HaLi Boyce, Ainsley E. Cogburn, Hazal Y. Gurcan, Brittany Matheson, Nandini Datta, James Lock

**Affiliations:** Department of Psychiatry and Behavioral Sciences, Stanford University School of Medicine, Stanford, CA, United States

**Keywords:** avoidant/restrictive food intake disorder, family meal, family-based treatment, mealtimes, parental comments, parent-child relationship

## Abstract

**Objective:**

Avoidant/restrictive food intake disorder (ARFID) is characterized by an avoidance or restriction of food intake due to a lack of interest in eating or foods, sensory sensitivities, and/or fear of adverse consequences, resulting in physical, medical, or psychosocial impairment. While previous studies in other eating disorders have found correlations between parental hostility, critical comments, and treatment outcomes, the ways that parents display their emotions and thoughts have not been evaluated in families of children with ARFID.

**Method:**

This study coded parental emotions and responsiveness in therapist-guided family meals during session two of manualized Family-Based Treatment (FBT) for ARFID to examine what types of comments were made. Thirty-one recorded meals were randomly selected from a randomized clinical treatment trial for children 6–12 years old with low-weight ARFID receiving FBT. The videos were double-coded with consensus and scoring guidelines developed in alignment with expressed emotion constructs (warmth, positive comments, hostility, critical comments, and emotional overinvolvement) but modified to fit the age range of ARFID children and the setting of the family-meal.

**Results:**

The study found that parents tended to make more positive comments than critical comments, with low scores of hostile comments and emotional overinvolvement.

**Discussion:**

Overall, these findings may suggest that some families enter treatment with relational strengths that can be leveraged therapeutically to reduce mealtime conflicts and improve treatment outcomes. The findings also support the establishment of a coding framework for parental emotions during mealtimes of families of children with low-weight ARFID and highlight the need for further research.

## 1 Introduction

Avoidant/restrictive food intake disorder (ARFID) is characterized by a lack of interest in eating or foods, sensory sensitivities, and/or a fear of aversive consequences resulting in physical, medical, or psychosocial impairment (1, 2). In youth, prevalence of ARFID is estimated to be between 0.3% and 15.5% (3). Children with ARFID exhibit more behavioral problems than children without eating difficulties during mealtimes such as greater food fussiness, less food engagement, and more reliance on parent feeding when compared to children without eating difficulties (4–6). Consequently, parents sometimes struggle with balancing mealtime behavior management with ensuring adequate intake (7). The relationship between parents and children during mealtimes are important, as negative interactions and mealtime stress are associated with disordered eating behaviors in children (8). Parents with high “expressed emotion” (EE) are defined as having parent-child interactions characterized by high criticism, hostility, and emotional overinvolvement (9–11). In adolescent and adult eating disorder populations such as anorexia nervosa (AN) and bulimia nervosa (BN), high parental EE is related to poorer treatment outcomes and greater eating disorder symptom severity (12–14). However, the types of emotions that parents display in response to their children with ARFID at mealtimes have not been systematically studied.

Family-based treatment for ARFID (FBT-ARFID) is an empirically supported treatment aimed at empowering parents to renourish their child and reduce the severity of ARFID behaviors (15–17). The treatment focuses on externalizing ARFID by separating the symptoms/behaviors of the disorder from the patient, thereby allowing parents to address the disorder while minimizing direct criticisms of the child (15). In the first session of FBT-ARFID, families are oriented to the treatment, provided psychoeducation on ARFID and its medical and psychosocial impacts, and parents are charged with the task of helping renourish their child (15). Families are also given instructions about the family meal to occur at the following session. Then, during the second session of FBT-ARFID, therapists conduct a family meal to help parents learn how to support and manage challenges with eating behaviors. Importantly, FBT does not include or support parents force feeding their child (15). This report describes findings from an exploratory study examining the types of comments and emotions that parents displayed about their children during this mealtime session. Based on studies of EE in AN, we predicted that parents would express greater positive (warmth and support) comments compared to negative (criticism and hostility) ones.

## 2 Materials and methods

Prior to enrolling, participants completed informed consent (parents/guardians) and assent (child participants) forms from a protocol approved by the IRB of our institution.

Videos were coded using a framework derived from the standardized EE codebook (10, 11, 18) but modified to fit the population and modality (session 2 FBT-ARFID family meal) as follows.

### 2.1 Coding scheme

#### 2.1.1 Positive comments

Positive Comments were defined as any positive comments about the child or their behaviors, specifically including positive reinforcement or praise, compliments about the child's character, or acknowledgements and support about the child's struggles. Scores were calculated as a total count of positive statements during the session. Coders counted the number of positive statements parents made to their child or to another individual present for the meal, such as a sibling or the therapist, about the child.

#### 2.1.2 Critical comments

Critical Comments were defined as any negative or critical comments that parents made to the child about their behavior during mealtimes, towards foods, and/or the child's ARFID symptoms in general. Coders counted the number of critical statements parents made to their child or to another individual present for the meal about the child.

#### 2.1.3 Hostile comments

Hostile Comments were distinguished from critical comments about eating behaviors and thus were defined as any negative statement to the child or generally about the child outside of the context of mealtimes and the child's ARFID. Coders counted the number of hostile statements parents made to their child or to another individual present for the meal about the child.

#### 2.1.4 Warmth

Warmth was rated based on how endearing, sympathetic, and positive the overall tone of parents was during the meal. Rather than a count of the number of statements, warmth was coded as a global score on a scale of 0–5, with 0 being no warmth and 5 being very warm.

#### 2.1.5 Emotional overinvolvement

Emotional Overinvolvement (EOI) was scored on a scale of 0–5, with 0 as no EOI and 5 as exaggerated and/or many emotional instances. A global score was calculated based on parental displays of intense emotional responses about the child, the eating disorder, eating behaviors, and/or parental difficulty with emotional regulation.

### 2.2 Analytic approach

Three coders (authors HB, AC, and HG) double-coded 31 randomly selected family meal session therapy videos of children 6–12 years old with ARFID receiving FBT-ARFID (17; NCT0377821). Mothers were usually designated Caregiver 1, resulting in Caregiver 1 being predominantly female (*n* = 30: 96.8%). Fathers were usually designated Caregiver 2, resulting in these being predominantly male (*n* = 28: 96.6%). In households with two caregivers, caregivers were coded as a dyad, with both parents contributing to the family's mealtime scores.

Inter-rater reliability scores were assessed through Intra-class Correlation Coefficients (ICC) based on a mean-rating (k = 2) as a two-way random-effects model with absolute agreement prior to consensus coding (19). The ICC estimates and their 95% confidence intervals are as follows: Positive Comments [0.70, CI = (0.47, 0.84)], Critical Comments [0.56, CI = (0.26, 0.76)], Hostile Comments [0.89, CI = (0.77, 0.94)], Warmth [0.77, CI = (0.57, 0.88)], and EOI [0.58, CI = (0.30, 0.77)]. Due to the wide range of agreement across the items, the authors met with a third-party mediator (BM) to resolve discrepancies and reach consensus. The consensuses codes were then used in the analysis of the data.

## 3 Results

Please see Table 1 for full details about the demographic characteristics of the participants. Briefly, 83.9% were dual-parent households, 71% of families had at least one sibling in the household, and 88.3% of children self-identified as white. Overall, caregivers identified almost evenly between female (*n* = 31; 51.7%) and male (*n* = 29; 48.3%) and had a mean age of 43.66 years old (SD = 6.8); however, one Caregiver 2 did not report their age. Many caregivers had completed graduate/professional school [Caregiver 1: *n* = 18 (58.1%); Caregiver 2: *n* = 11 (37.9%)]. Children were predominantly male (*n* = 22; 71%) with a mean age of 9.45 years old (SD = 2.1). The mean age of ARFID onset was 3.2 years old (SD = 3.0). The mean overall ARFID severity score was 2.9 (SD = 0.7) before the start of treatment, which was assessed by the Pica, ARFID, Rumination Disorder Interview completed by parents (20). Additionally, 19 (61.3%) of the children participants had an additional comorbid psychiatric disorder (61.3%).

**Table 1:** Participant demographics.

Variable	Child (*n* = 31)	Caregiver 1 (*n* = 31)	Caregiver 2 (*n* = 29)
Age (Years)			
Mean (standard deviation)	9.45 (2.1)	42.94 (6.5)	44.46 (7.2)^a^
Sex, *N* (%)			
Female	9 (29)	30 (96.8)	1 (3.4)
Male	22 (71)	1 (3.2)	28 (96.6)
Ethnicity, *N* (%)			
Hispanic	5 (16.1)	4 (12.9)	3 (10.3)
Non-Hispanic	26 (83.9)	27 (87.1)	26 (89.7)
Race, *N* (%)			
American Indian or Native Alaskan	1 (3.2)	–	1 (3.4)
Asian	5 (16.1)	3 (9.7)	5 (17.2)
Black or African American	1 (3.2)	–	2 (7)
More than one race	5 (16.1)	3 (9.7)	–
White	19 (61.3)	25 (80.6)	21 (72.4)
Family Type, *N* (%)			
Intact	26 (83.9)	–	–
Divorced	3 (9.7)	–	–
Other/ No Response	2 (6.5)	–	–
Number of Siblings, *N* (%)			
None	9 (29)	–	–
One	13 (41.9)	–	–
Two	8 (25.8)	–	–
Three	1 (3.2)	–	–
Parent Education, *N* (%)			
Graduated high school	–	1 (3.2)	5 (17.2)
Partial completion of college	–	2 (6.5)	1 (3.4)
Graduated from 2-year college	–	2 (6.5)	–
Graduated from 4-year college	–	8 (25.8)	9 (31)
Partial completion of graduate/professional school	–	–	3 (10.3)
Completion of graduate/professional school	–	18 (58.1)	11 (37.9)
Child Comorbid Psychiatric Disorders, *N* (%)			
At least one comorbid disorder	19 (61.3)	–	–
Anxiety Disorders	7 (22.6)	–	–
Attention-Deficit/ Hyperactivity Disorder	10 (32.3)	–	–
Obsessive-Compulsive Disorder	1 (3.2)	–	–
Major Depressive Disorder	1 (3.2)	–	–
Tic/Tourette Symptom	4 (12.9)	–	–

a One Caregiver 2 declined to provide age.

### 3.1 Positive comments

Most families made at least one positive comment (*n* = 28; 90.3%) with an average positive comment count score of 4.16 (SD = 3.37). Positive comments included praise, encouragement or acknowledgement of eating difficulties such as “Good/Great Job!”, “You can do it!”, “I'm proud of you!”, and “I'm so impressed”. Some parents applauded their child's efforts, commenting, “way to eat past that point of fullness” or “you still finished [meal], even though you didn't like it and it was great!”.

### 3.2 Critical comments

Critical comments were identified less frequently than positive comments, with most families making at least one critical comment (*n* = 24; 77.42%) and an average count score of 2.32 (SD = 2.59). Three family meals (9.7%) contained more than five critical comments. Most critical comments reflected a caregiver's frustration or minimization of the child's experience as follows: “I can clean the whole house, and he's still eating”, “think about how much further we would be if we didn't have to negotiate this long”, and “even [the child's] normal time [for eating] is too long”. Frustration with eating behaviors were expressed as follows: “No room? You haven't eaten anything!”, “Aside from doughnuts…that's not even really a meal”, “He'll only have a bite, he won't have it as a meal”, and “You asked for this so why aren't you eating it?”.

### 3.3 Hostile comments

Broad hostile comments unrelated to ARFID behaviors were infrequently observed. Only three videos received a hostility rating greater than zero, resulting in an average hostility score of 0.29 (SD = 1.27). Hostile comments included statements such as “he's raging about something every day, it's always something” and “I've heard a lot of excuses from you over the years, over a wide variety of things, including eating … it's never ‘oh, okay,’ it's always an excuse.”

### 3.4 Warmth

Mealtimes videos generally had high scores for warmth, with many families scoring a 4 on the global scale (*n* = 14; 45.16%), resulting in an average score of 3.26 (SD = 0.93) (Table 2). Comments contributing to warmth included compliments such as “You do incredible things” or “He's pretty adventurous”. Physical encouragement also contributed to warmth scores, including behaviors like rubbing their child's back or giving a high five after an eating task.

**Table 2:** Emotional commentary subscales for parents at family meal.

Subscale	Mean	(SD)	Range
Emotional over-involvement *(Possible Range: 0–5)*	0.39	(0.67)	0–2
Warmth *(Possible Range: 0–5)*	3.26	(0.93)	1–4
Criticism *(Count Score)*	2.32	(2.59)	0–13
Positive comments *(Count Score)*	4.16	(3.37)	0–13
Hostility *(Count Score)*	0.29	(1.27)	0–7

### 3.5 Emotional overinvolvement

Average EOI was low, with most families (*n* = 22; 71%) scoring 0, and no family scoring above 2. The mean EOI in the sample was 0.39 (SD = 0.67). Higher EOI scores were characterized by overly accommodating behaviors or parental emotional dysregulation related to the child's ARFID. For instance, some parents described closely monitoring family conversations in the hours before meals to prevent their child from becoming irritated and “taking it out on the food.” Other parents became emotionally dysregulated when discussing their child's eating difficulties, leading to arguments between parents or children.

## 4 Discussion

This exploratory study assessed the types of comments that parents of children with ARFID made during the family meal session of FBT-ARFID. Overall, 90.3% of the caregiver dyads were warm and made positive comments during the meal. Parents generally refrained from hostile comments, with 87.1% not making any hostile comments. In addition, few parents demonstrated EOI at mealtime, with only 9 of the 31 families scoring above a 0. These results share similarities with previous studies of EE in families with children with eating disorders where parents tended to be warmer and more positive than critical and hostile toward their children (21, 22).

### 4.1 Clinical importance

The therapist-guided family meal is a critical session in manualized FBT-ARFID, offering a real-time opportunity to guide parents in supporting behavioral change and renourishment when needed. This study provides the first description of parental emotions during the family meal session in parents of children with ARFID. The findings suggest that most families enter treatment with relational strengths that can be leveraged therapeutically to reduce mealtime conflicts and potentially improve outcomes, as have been found in previous studies examining AN (23–25). The types of critical comments that were identified illustrate common frustrations related to difficulties with parental attempts to manage eating behaviors in children with ARFID such as negotiations, accommodations, or minimization of the child's distress (7, 25, 26). FBT-ARFID aims to help parents better manage eating behaviors and reduce conflict around mealtimes by understanding the child's difficulties as a manifestation of their disorder, with a goal of having the child be able to eat to support their psychosocial functioning and growth needs. Therefore, the critical comments are potential areas therapists might identify for targeted coaching using key components of FBT-ARFID related to agnosticism and externalization to thereby decrease guilt and blame towards the child, the parents, or both. Future research should examine whether patterns of parental meal-time positive or critical comments or family environment (warmth, EOI, or hostility) may influence the parent-child relationship and the processes that are associated with treatment outcomes. Future research should also examine child behaviors and emotions in relation to the parent comments, the parent-child relationship, and treatment outcomes.

### 4.2 Strengths, limitations, and future directions

This study's strengths include double coding among three raters and a coding scheme modeled on an established coding scheme. Another strength of the study is that the sample consisted of participants randomly selected from a systematic study FBT-ARFID. None of the coders or research team on the project provided treatment in the study. Author JDL provided supervision while the research trial was ongoing, however he was not involved in the coding or analysis of the data. One of the study's limitations is the varying interrater reliability of the items between the coders. To address this, a consensus was reached with a mediator. Additionally, the data were collected within the setting of the FBT therapist-directed family meal. As therapists use the family meal to gain insight into family functioning and the child's eating, they may prompt parents to discuss difficulties surrounding their child's eating or praise the child for their efforts, influencing what parents say during the session. Additionally, the Hawthorne effect may have influenced study results as parents may have changed their behaviors in the presence of a therapist observer. Therefore, the comments and emotional tone parents exhibit during the session may not generalize to what typically occurs at mealtimes at home.

Further studies are needed to examine the types of emotions that parents may have about their child and their child's ARFID, including considering studies employing standard EE procedures. It would also be important to determine whether parental emotions and expressions impact outcomes in FBT-ARFID. More broadly, a better understanding of the emotional milieu of families with children with ARFID could potentially identify new treatment targets or therapeutic approaches.

## Data Availability

The raw data supporting the conclusions of this article will be made available by the authors, without undue reservation.
